# Fungal *Shaker*-like channels beyond cellular K^+^ homeostasis: A role in ectomycorrhizal symbiosis between *Hebeloma cylindrosporum* and *Pinus pinaster*

**DOI:** 10.1371/journal.pone.0242739

**Published:** 2020-11-20

**Authors:** Kevin Garcia, Carmen Guerrero-Galán, Hannah E. R. Frank, Muhammad Zulqurnain Haider, Amandine Delteil, Geneviève Conéjéro, Raphaël Lambilliotte, Cécile Fizames, Hervé Sentenac, Sabine D. Zimmermann

**Affiliations:** 1 Department of Crop and Soil Sciences, North Carolina State University, Raleigh, California, United States of America; 2 BPMP, Université de Montpellier, CNRS, INRAE, Institut Agro, Montpellier, France; 3 Plateforme Histocytologie et Imagerie Cellulaire Végétale, INRA-CIRAD Montpellier, France; Friedrich Schiller University, GERMANY

## Abstract

Potassium (K^+^) acquisition, translocation and cellular homeostasis are mediated by various membrane transport systems in all organisms. We identified and described an ion channel in the ectomycorrhizal fungus *Hebeloma cylindrosporum* (*Hc*SKC) that harbors features of animal voltage-dependent *Shaker*-like K^+^ channels, and investigated its role in both free-living hyphae and symbiotic conditions. RNAi lines affected in the expression of *HcSKC* were produced and used for *in vitro* mycorrhizal assays with the maritime pine as host plant, under standard or low K^+^ conditions. The adaptation of *H*. *cylindrosporum* to the downregulation of *HcSKC* was analyzed by qRT-PCR analyses for other K^+^-related transport proteins: the transporters *HcTrk1*, *HcTrk2*, and *HcHAK*, and the ion channels *HcTOK1*, *HcTOK2*.*1*, and *HcTOK2*.*2*. Downregulated *HcSKC* transformants displayed greater K^+^ contents at standard K^+^ only. In such conditions, plants inoculated with these transgenic lines were impaired in K^+^ nutrition. Taken together, these results support the hypothesis that the reduced expression of *HcSKC* modifies the pool of fungal K^+^ available for the plant and/or affects its symbiotic transfer to the roots. Our study reveals that the maintenance of K^+^ transport in *H*. *cylindrosporum*, through the regulation of *HcSKC* expression, is required for the K^+^ nutrition of the host plant.

## Introduction

Potassium (K^+^) is an essential cation involved in many biological processes in plants, such as growth, photosynthesis and stress tolerance [1,2]. However, most soil K^+^ cations are complexed with minerals such as feldspars or micas, resulting in low mobility and availability. As a consequence, productivity of agricultural and agroforestry ecosystems is strongly dependent on large and regular addition of chemical fertilizers [3]. Plants developed various strategies to overcome this lack of K^+^ availability and to improve its uptake, such as expression of high-affinity transport systems, exudation of organic acids by the root system, and symbiotic associations with soil-living microbes (reviewed in [4–7]).

Ectomycorrhizal (ECM) symbiosis is an intimate relationship between the roots of woody plants and the mycelium of soil-borne fungi, and constitutes a major component of boreal and temperate forest ecosystems [8]. The main benefit of this association for the host plants is the improvement of macro- and micronutrient acquisition [9]. In ECM structures, nutrients are transferred from the soil-exploring extraradical mycelium to the hyphae developing *in planta*, through a fungal sheath surrounding the roots. The intra-radical colonization by the fungus forms a specific structure called the Hartig net. This net develops between the host root epidermis and cortex, where various transport proteins are expressed to drive nutrient exchanges between the two partners [9,10]. While most reports have focused on nitrogen and phosphorus nutrition in ECM plants, some studies have reported a significant transfer of fungal K^+^ towards the host, particularly in K^+^-limited and stressful conditions [11,12]. One hypothesis is that the ectomycorrhiza-mediated K^+^ nutrition could also be a strategy for the host plant to alleviate salt stress responses [13]. The transfer of K^+^ through the mycorrhizal pathway requires the expression and organization of fungal transport proteins specific to the soil-fungus or plant-fungus interfaces, allowing K^+^ movements from the soil into colonized roots. The fine tuning of this putative fungal hyphae polarization seems to be required for the distribution of K^+^ from the soil to plant cortical apoplasm [4].

Several proteins from ECM fungi involved in plant nutrient allocation have been identified and characterized [9,14–16], and some of these might be responsible for the maintenance of ECM symbiosis, in addition to exclusive involvement in trophic exchanges [17]. To our knowledge, only a handful of transport proteins involved in K^+^ transport has been reported in ECM fungi thus far. Among them, *Hc*Trk1 from *Hebeloma cylindrosporum* [18] is specifically localized in extra-radical hyphae of *H*. *cylindrosporum-Pinus pinaster* ectomycorrhizas, suggesting a role in K^+^ uptake from the soil [12]. Genome sequencing of *H*. *cylindrosporum* [19] has allowed the identification of other putative transporters possibly involved in K^+^ acquisition from the soil, *Hc*Trk2 and *Hc*HAK [4], but their roles remain to be elucidated. Recently, we also described for the first time three fungus-specific tandem-pore outwardly rectifying K^+^ (TOK) channels from *H*. *cylindrosporum* [20,21]. Excitingly, we were able to use an overexpression approach to demonstrate that one of them, *Hc*TOK2.2, participates in the release of K^+^ towards colonized *P*. *pinaster* roots.

In plants and animals, the efflux of K^+^ from cells is a crucial biological process involved in the electrical polarization and energization of the plasma membrane, as well as in adaptation to biotic and abiotic stresses. Potassium channels and non-selective cation channels are involved in such mechanisms [22]. The large family of tetrameric voltage-dependent K^+^ channels (K_V_), often called *Shaker*-like channels, constitutes a key component of the plasma membrane conductance. Many members are major contributors to the voltage-dependent influx or efflux of K^+^ in plants and animals [23–25]. To our knowledge, although some *Shaker*-like channels were identified *in silico* in ECM fungi, none has been studied so far [4]. Previous evidence in plants and animals establishes the relevance of this type of channels in the regulation of cellular K^+^ homeostasis, and suggests fungal *Shaker*-like channels are likely to have a similar function. Moreover, they are possible candidates for K^+^ allocation from ECM fungi towards the host plant at the symbiotic interface and/or K^+^ storage into the vacuole.

In this work, we provide the first description of the fungal *Shaker*-like K_V_ channel family by *in silico* and phylogenetic analyses, and investigate the physiological role of one of them, *Hc*SKC from *H*. *cylindrosporum*. To assess the role of this channel in the allocation of fungal K^+^ to the host plant *P*. *pinaster*, we generated transgenic fungal lines downregulated in *HcSKC* expression. We demonstrate that *HcSKC* silencing affects the fungal K^+^ homeostasis, alters the expression of other fungal K^+^ transport systems, and attenuates the K^+^ acquisition of ECM *P*. *pinaster*.

## Materials and methods

### Wild-type and transgenic fungal strains

The homokaryotic strain h7 of the ECM Basidiomycota *H*. *cylindrosporum* Romagnesi [26] was grown in the dark at 26°C in YMG medium (Yeast extract, Malt extract, Glucose) [27] either on agar-solidified Petri dishes or in liquid cultures.

The *Agrobacterium tumefaciens-*mediated genetic transformation process and production of the empty vector fungal strain (pPZP-133 control) have been detailed previously [12]. Using the same protocol and the bacterial strain LBA1126, carboxin-resistant RNAi-SKC fungal lines were produced.

### Construction of RNAi-SKC plasmids

A 320 bp *HcSKC*-specific region was amplified on cDNA using RNAi-specific primers (S1 Table) for the construction of the silencing vector. The sense (S) and antisense (AS) fragments obtained were inserted into the two MCS of pSILBAγ vector used for gene silencing in *Laccaria bicolor* [28]. The silencing cassette was digested using *Xba*I enzyme and inserted in pPZP-133 plasmid in order to get the pPZP-RNAi-SKC vector.

### Ectomycorrhiza production

Maritime pine seeds (*P*. *pinaster* Soland in Ait. from Medoc, Landes-Sore-VG source, France) were sterilized with 37% H_2_O_2_ [29] and sown on Petri dishes containing agarose (Eurobio Molecular Biology Grade) and 0.2% glucose. The co-culture method in glass tubes as well as the composition of standard K^+^ (SK, 1 mM K^+^) and low K^+^ (LK, 0.05 mM K^+^) liquid N1 media were described previously [12]. Culture conditions were 16 h-photoperiod (210 μmol.m^-2^.s^-1^), 20°C and 60% relative humidity. SK medium was used for *in situ* hybridization experiments, and both SK and LK media for assays analyzing the ECM phenotype of RNAi-SKC fungal lines. Six 2-month-old plants per condition were collected for each experiment.

### *In situ* hybridization

Two successive PCR amplifications of *HcSKC* cDNA led to the synthesis of sense and antisense probes (300 bp). The first amplification was performed with ISHSKC-T7-S-F/ISHSKC-S-R or ISHSKC-AS-F/ISHSKC-T7-AS-R primers (S1 Table). A 1:100 dilution of the first PCR product was used for the second amplification with ISHT7-Prom and specific SKC-F or -R primers. Ectomycorrhizas were embedded in paraffin and 8 μm sections were obtained using a microtome. Sample preparation and hybridization were performed as previously described [20,30]. Slides were observed with a Leica DM6000 wide-field microscope (Montpelier RIO Imaging platform, www.mri.cnrs.fr) and pictures were analyzed by Volocity Acquisition 5.1.0 software (Perkin Elmer, www.perkinelmer.com).

### Potassium shortage in *Hebeloma cylindrosporum* pure culture

Empty vector and RNAi-SKC fungal strains were cultivated in N6 liquid medium [31] containing 10 mM K^+^ (6 mM KNO_3_, 4 mM KCl). Fresh N6 was supplied on days 7 and 12. On day 14, thalli were washed five times with N6 medium without added K^+^ and cultivated in these media up to 48 h. In medium without K^+^, NO_3_ was added in the form of Ca(NO_3_)_2_ at the same concentration as in the standard K^+^ medium. Fungi were collected 0, 12, 24 and 48 h after K^+^ deprivation and washed twice in CaSO_4_ (0.2 mM)–glucose (5 gl^-1^) solution. Half of each sample was flash-frozen in liquid nitrogen for qRT-PCR analyses and the rest was dried for one week at 60°C, weighed and used for intracellular K^+^ content determination.

Additionally, four media with high or low concentrations of K^+^ (1 or 0.05 mM, respectively) and sodium (Na^+^; 1 or 0.2 mM, respectively) were used to assess fungal biomass of transgenic strains (S2 Table). These media were named: +K/-Na, +K/+Na, -K/-Na, and -K/+Na. Transgenic fungal strains were cultured on a solid version of these media for 1 week at 26°C, sub-cultured into 50 ml of their corresponding liquid media and placed at 26°C for 28 days. Thalli were collected, dried at 70°C, and dry weights were determined.

### Quantification of potassium contents

Plant tissue and mycelia were collected and weighed to determine the dry biomass (DW). Acid extraction of tissue ion contents and assays of K^+^ content by flame atomic absorption spectrophotometry were performed as previously described [12].

### qRT-PCR analyses

*H*. *cylindrosporum* RNA extraction, cDNA synthesis and qRT-PCR protocol were described [30]. Expression levels of the transport systems *HcSKC* (protein ID 79961; https://mycocosm.jgi.doe.gov/Hebcy2/Hebcy2.home.html), *HcTOK1* (31571), *HcTOK2*.*1* (129509), *HcTOK2*.*2* (127201), *HcTrk1* (445173), *HcTrk2* (176376), and *HcHAK* (435192) were determined relatively to the internal control α-tubulin (24108) on mycelium samples (for primers see S1 Table).

### *In silico* analysis of *HcSKC*

Hydrophobicity profiles of *Hc*SKC (*H*. *cylindrosporum*), *Lb*SKC (*L*. *bicolor*), and *Xl*K_V_2.1 (*Xenopus laevis*) subunits were obtained using Kyte-Doolittle algorithm with a window size of 11 amino acids [32] (http://gcat.davidson.edu/DGPB/kd/kyte-doolittle.htm).

Amino acid alignment of *Hc*SKC, *Lb*SKC, and *Xl*K_V_2.1 subunits was performed using Clustal Omega program (http://www.ebi.ac.uk/Tools/msa/) and formatted with BoxShade program (http://www.ch.embnet.org/software/BOX_form.html).

Homology structure of the *Hc*SKC subunit was modeled by Swiss-Model server using the template structure of *Rattus norvegicus Rn*K_V_2.1 subunit [33] (http://swissmodel.expasy.org/). Amino acid alignment between these two subunits was obtained using the same program.

### Phylogenetic tree construction

The protein sequences of SKC subunits of 185 fungi, 4 animals and 2 plants were collected from JGI [34], NCBI (NCBI Resource Coordinator 2015), UniProt (The UniProt Consortium 2015) or TAIR (www.arabidopsis.org) databases by BLASTP analysis based on *Hc*SKC of *H*. *cylindrosporum* (S2 Table). All fungal databases used here are publicly available. All sequences without a predicted G[YF]G[DE] pore motif were removed manually. Protein sequences were aligned with MUSCLE algorithm v3.8.31 [35] and the alignment was cured by Gblocks program [36]. Phylogenetic analysis was performed by PhyML [37] using the maximum likelihood method (1000 bootstraps) and the tree was visualized by Dendroscope software v3.2.10 [38].

### Construction of the *HcSKC-EGFP* fusion and expression in *Saccharomyces cerevisiae*

The *HcSKC-EGFP* C-terminal cassette was created with a PCR fusion approach, amplifying separately *HcSKC* from *H*. *cylindrosporum* cDNA and *EGFP* from a preexisting construct, with primers overlapping the fusion region containing both *HcSKC* and *EGFP* sequences (S1 Table). A third PCR with the flanking primers was required to amplify the whole construct, which was later digested with restriction enzymes and inserted in the corresponding vectors. Subcellular localization of the *Hc*SKC-EGFP construct in the yeast strain ply232 was attempted with two approaches, first a galactose-induced expression in the vector pYES2 (ThermoFisher Scientific) and later under the constitutive promoter PGK in the pFL61 vector [39].

### Statistical analyses

Data normality was checked using the Wilk-Shapiro test. Differences between averages were analyzed by one-way ANOVA followed by Tukey HSD post-hoc tests, or by Student’s test, depending on the experiment. Mean comparisons were carried out using Dunnett’s test. Dunnett’s test was also used to search for significant differences in pairwise comparisons. All statistical analyses were performed with R software at the 5% level of statistical significance.

## Results

### Identification of a *Shaker*-like K_V_ channel in *Hebeloma cylindrosporum*

**Fig 1:**
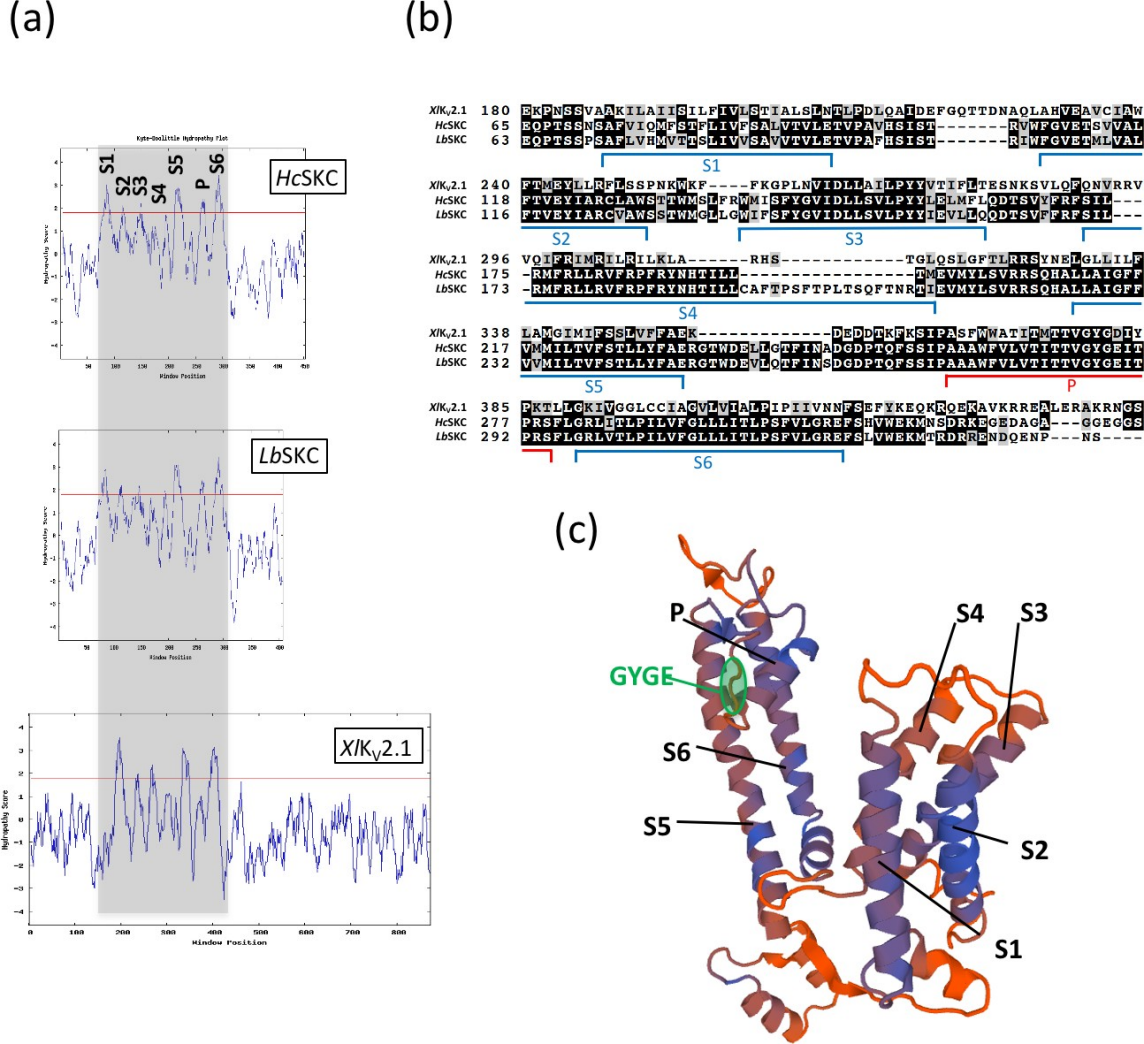
*In silico* prediction of a *Shaker*-like channel in the fungus *Hebeloma cylindrosporum*. The hydrophobicity profiles of the *Shaker* subunits from *H*. *cylindrosporum* (*Hc*SKC), *L*. *bicolor* (*Lb*SKC), and *X*. *laevis* (*Xl*K_V_2.1) (a) and their partial amino acid sequence alignment (b) predicted six transmembrane domains (S1 to S6). One pore domain in *Hc*SKC and *Lb*SKC subunits containing the conserved G[YF]G[DE] motif of the *Shaker* channel selectivity filter was also detected. The S4 transmembrane domain corresponds to the predicted voltage-sensor domain. (c) The plausible 3-D model of the fungal *Hc*SKC subunit showing the transmembrane (S1 to S6) and pore (P) domains was generated by homology with the 3-D structure of *Rn*K_V_2.1 K^+^ channel from *R*. *norvegicus* (NP_037318.1; S2 Table) as a template using Swiss-Model server [33] (http://swissmodel.expasy.org/). The GYGE signature motif of the pore region was highlighted in green. The sequence alignment of *Hc*SKC and *Rn*K_V_2.1 polypeptides is displayed in S1 Fig.

We previously identified a putative K^+^ channel (STC no.: 009A07R1.0A1.1) in EST resources obtained from *H*. *cylindrosporum* pure cultures [40]. BLASTP analysis of 009A07R1.0A1.1 protein sequence against the *L*. *bicolor* S238N genomic database allowed the identification of a unique orthologue (protein ID 297800; https://mycocosm.jgi.doe.gov/Lacbi2/Lacbi2.home.html). The hydrophobicity profile of these two proteins generated by the Kyte-Doolittle algorithm revealed the presence of 7 hydrophobic domains, similar to the *Xl*K_V_2.1 *Shaker* channel from *X*. *laevis* (Fig 1A). The amino acid alignment of these fungal *Shaker*-like proteins with *Xl*K_V_2.1 confirmed the presence of 6 conserved transmembrane domains and one pore domain characterized by the GYGE motif ensuring K^+^ selectivity (Fig 1B). Consequently, we named these proteins SKC (*Shaker*-like K^+^-Channel). The S4 domain (constituting the voltage sensor in *Shaker* channels) of *Hc*SKC and *Lb*SKC harbored several arginine residues (R). Such positively charged residues have been shown in other K_V_ channels to contribute to the channel voltage sensitivity and gating charge [41]. When compared with *Xl*K_V_2.1, *Hc*SKC and *Lb*SKC have a shorter C-terminal region, suggesting reduced "built-in" regulation mechanisms (Fig 1A). Based on these *in silico* analyses, we used the *R*. *norvegicus Rn*K_V_2.1 channel (NP_037318.1; S3 Table) as a template to propose a structural model for the fungal *Shaker*-like proteins (Fig 1C, S1 Fig).

### Distribution of *Shaker*-like channels in the fungal kingdom

**Fig 2:**
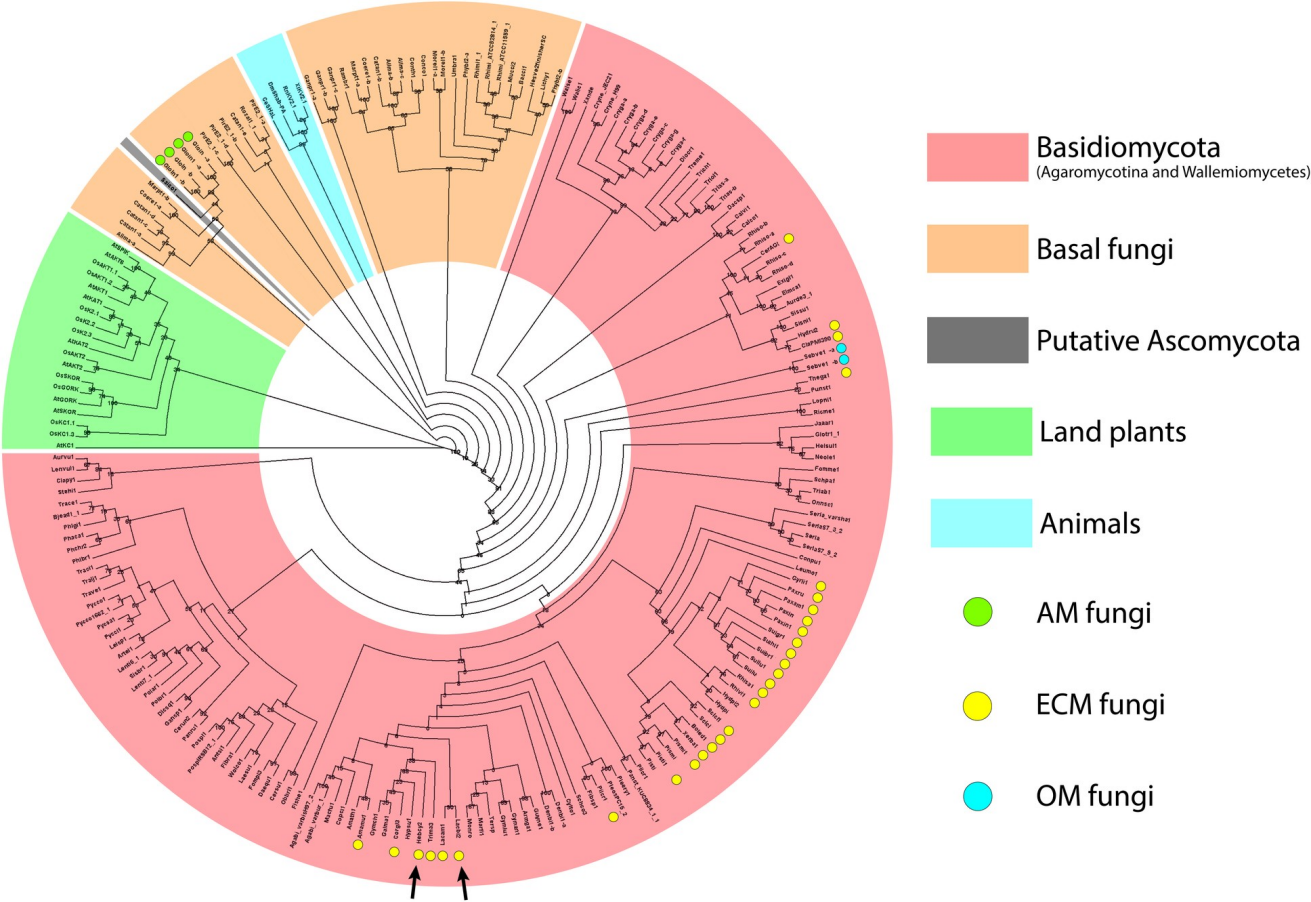
Phylogenetic tree of K_V_ channels in fungi, and representative animal and plant species. The *Shaker*-like K^+^ channel tree of publicly available sequenced fungi from the Basidiomycota phylum and from basal fungi (Cryptomycota, Chytridiomycota, Blastocladiomycota, Zoopagomycota and Mucoromycota) was calculated by the maximum likelihood method (1000 bootstraps). *Shaker*-like proteins were found only in one putative Ascomycota species (*Saitoella complicata*) and not in the Pucciniomycotina and Ustilaginomycotina subphyla of Basidiomycota. Ectomycorrhizal (ECM), arbuscular mycorrhizal (AM), and orchid mycorrhizal (OM) fungi are indicated by yellow, green, and blue dots, respectively. K_V_ channels from representative land plant and animal species were included in the tree. All species displayed in this tree are listed in the S2 Table. *Shaker*-like proteins from *H*. *cylindrosporum* (*Hc*SKC) and *L*. *bicolor* (*Lb*SKC) used in this study are indicated by black arrows.

Using *Hc*SKC as a reference, we performed BLASTP analysis on publicly available sequenced fungi found on NCBI and JGI fungal portals (http://genome.jgi-psf.org/programs/fungi/index.jsf). In addition, a cure based on the presence of a putative G[YF]G[DE] pore motif was achieved. In the Basidiomycota phylum, the presence of putative SKC proteins was detected only in the Agaricomycotina subphylum, as is defined in the JGI phylogenetic tree (Fig 2). In addition, putative SKC proteins were found in *Wallemia melicola*, which belongs to Wallemiomycetes, a sister group to Agaricomycotina [42]. Two putative *Shaker*-like channels were also detected in the arbuscular mycorrhizal (AM) fungus *Rhizophagus irregularis* (Glomeromycotina subphylum) and other basal fungi belonging to Cryptomycota, Chytridiomycota, Blastocladiomycota, Zoopagomycota and Mucoromycota phyla (Fig 2). Surprisingly, no SKC was found in the Pucciniomycotina and Ustilaginomycotina subphyla of Basidiomycota, and in the Ascomycota phylum. Moreover, proteins similar to K_V_ channels, but without the G[YF]G[DE] motif, were not found in these fungi, indicating the complete loss of *Shaker*-like genes in Pucciniomycotina, Ustilaginomycotina and Ascomycota species, with the exception in the Ascomycota phylum of *Saitoella complicata*, which presents one putative SKC channel (Fig 2).

### The functional properties of *Hc*SKC could not be revealed in various heterologous systems

Functional characterization is an important step for the analysis of K^+^ channel activity to predict transport features, such as selectivity and direction of rectification. Many strategies were applied to characterize *Hc*SKC, using multiple heterologous systems (*X*. *laevis* oocytes and yeast) and co-injection of putative regulatory proteins (protein kinase K and putative channel β-subunits from *H*. *cylindrosporum*: *Hc*KCNAB1 and *Hc*KCNAB2) (S4 Table). No significant result was obtained from any of these experiments. Moreover, the expression of *Hc*SKC triggers endogenous currents in *X*. *laevis* oocytes, making the observation of specific exogenous currents tricky (S4 Table). In addition, the previously identified *Hc*SKC channel in the *H*. *cylindrosporum* h1 strain [40] has been extensively tested by voltage-clamp experiments in *X*. *laevis* oocytes, without any conclusive result (S4 Table). As an alternative, a functional characterization of the homologous channel in *L*. *bicolor Lb*SKC was also attempted in *X*. *laevis* oocytes, but unsuccessfully (S4 Table). Finally, attempts to perform subcellular localizations in yeast using *HcSKC-EGFP* fused constructs driven by a constitutive or a galactose-inducible promoter failed too (S2 Fig). None of the experiments yielded a clear localization pattern of *Hc*SKC, probably due to misprocessing of the protein product, reinforcing the issues in expressing *HcSKC* in the tested heterologous systems.

### *HcSKC* transcripts are localized in all types of ectomycorrhizal hyphae

**Fig 3:**
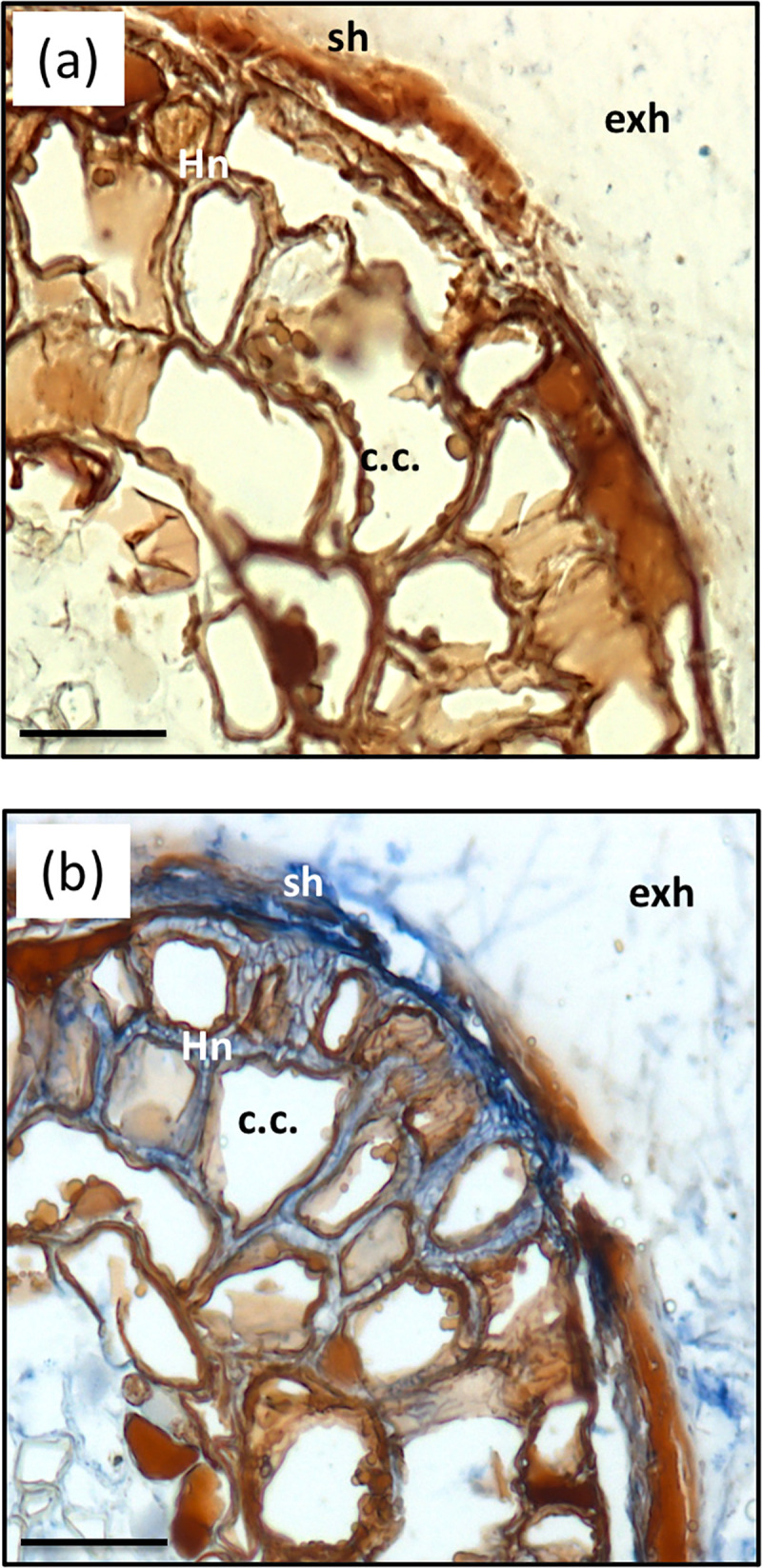
*HcSKC* transcript localization in *Hebeloma cylindrosporum*—*Pinus pinaster* ectomycorrhizas. *In situ* hybridization in ectomycorrhizas from 2-month-old co-cultures of *P pinaster*–*H*. *cylindrosporum* with *HcSKC*-specific probes. (a) Control with a sense probe did not show any signal. (b) Using the antisense probe, *HcSKC* transcripts were detected in extra-radical hyphae, the fungal sheath and the Hartig net of ectomycorrhizas. Bars, 50 μm. c.c., cortical cell; exh, extraradical hyphae; Hn, Hartig net; sh, fungal sheath.

To elucidate the role of *Hc*SKC channels in the symbiotic association with *P*. *pinaster*, the localization of corresponding transcripts was analyzed in 2-month-old ectomycorrhizas through *in situ* hybridization experiments. Cross-section and probe hybridization processes resulted in the detection of *HcSKC* transcripts in extra-radical hyphae, the hyphal mantle, and the Hartig net, indicating no specific expression of this channel in ectomycorrhizas (Fig 3). Only the antisense probe provided signals indicating the presence of *HcSKC* transcripts (Fig 3B). Since the sense probe sequence is not complementary to the *HcSKC* mRNA sequence, it cannot hybridize, and, therefore, serves as a negative control. Consequently, it did not give rise to any signal (Fig 3A). A construct harboring the *EGFP* gene marker fused to the *HcSKC* promoter was cloned to show that *HcSKC* was well expressed in free-living conditions (S3 Fig).

### Silencing of *HcSKC* affects *Hebeloma cylindrosporum* potassium homeostasis

**Fig 4:**
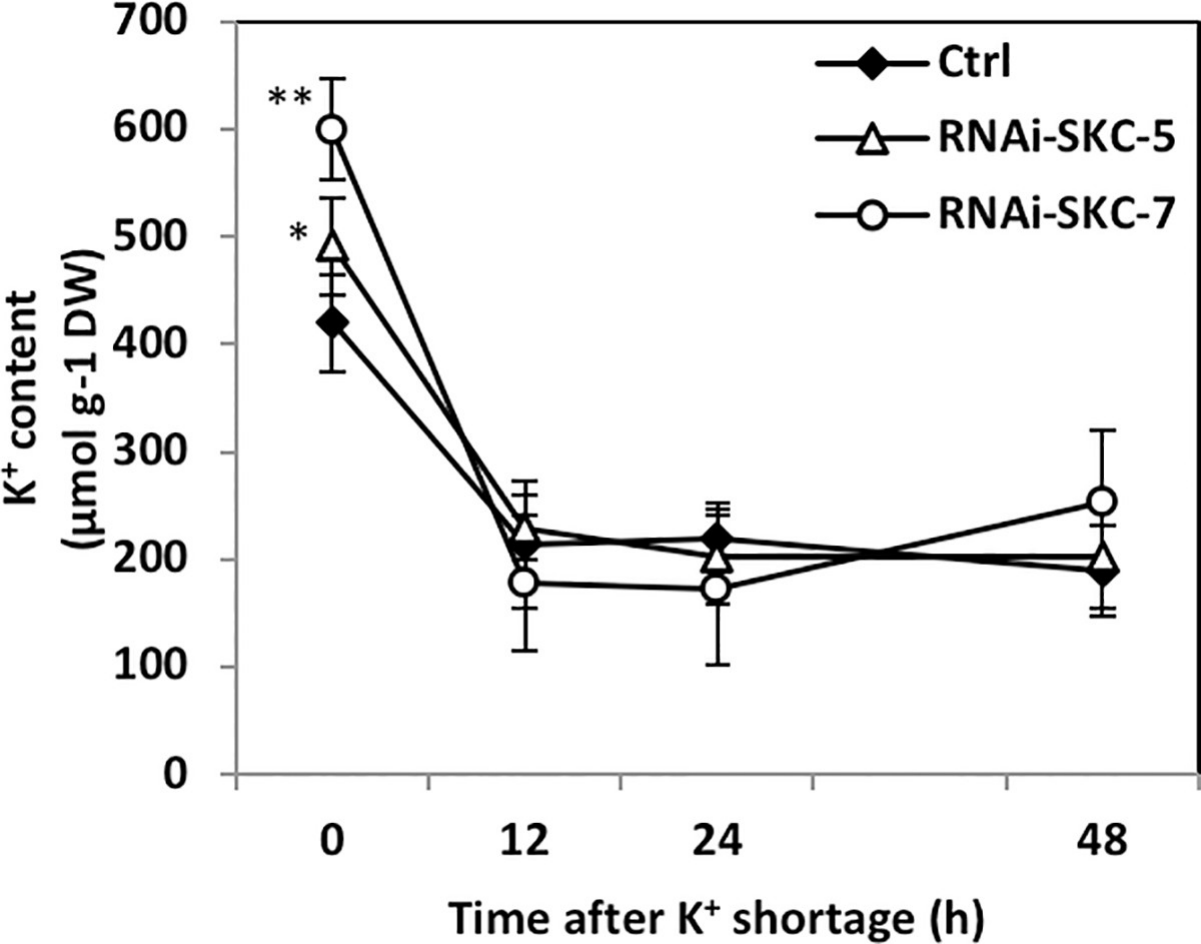
Potassium content in *Hebeloma cylindrosporum HcSKC* downregulated lines. K^+^ content was measured in fungal lines transformed with the empty (Ctrl) or *HcSKC*-silencing vectors (RNAi-SKC-5 and RNAi-SKC-7) using an atomic absorption flame spectrophotometer. Statistical differences were evaluated using the Student’s test with respect to the Ctrl strain (*, P < 0.05; **, P < 0.01). n = 6.

Decoding the role of *Hc*SKC in axenic conditions was attempted by RNAi downregulation of the corresponding gene. *HcSKC*-silencing lines of *H*. *cylindrosporum* were produced by *Agrobacterium tumefaciens–*mediated transformation. The expression level of *HcSKC* was determined by qRT-PCR in fungal mycelia growing on selection medium. Two isolates displaying a five-fold decrease in *HcSKC* expression, named RNAi-SKC-5 and RNAi-SKC-7, were selected for ECM assays (S4 Fig). We then investigated the K^+^ accumulation and expression of K^+^ transport-related genes in RNAi-SKC-5 and RNAi-SKC-7 lines. In fungi grown in standard medium (i.e., in presence of a relatively high external K^+^, 1 mM), internal K^+^ content was significantly higher in RNAi-SKC transformants than in the control line, indicating an increase in K^+^ acquisition and/or a decrease in K^+^ efflux (Fig 4). Once these fungal isolates were transferred into the K^+^-free medium, no difference in K^+^ content between RNAi-SKC and control lines could be detected at 12, 24 and 48 h after the transfer (Fig 4). In addition, we assessed the effect of high and low K^+^ (1 and 0.05 mM, respectively) and Na^+^ (1 or 0.2 mM, respectively) availability on fungal growth in all transgenic strains (S5 Fig). All media were prepared on the basis of the media used for the subsequent ECM assay (S4 Table). After 28 days of culture in axenic condition, dry weights were recorded. Although the biomass of both RNAi-SKC transgenic lines differed in all conditions, they did not significantly differ from the control line, except for RNAi-SKC-5 that showed a slightly reduced biomass in +K/-Na condition compared to the control line (S5 Fig).

**Fig 5:**
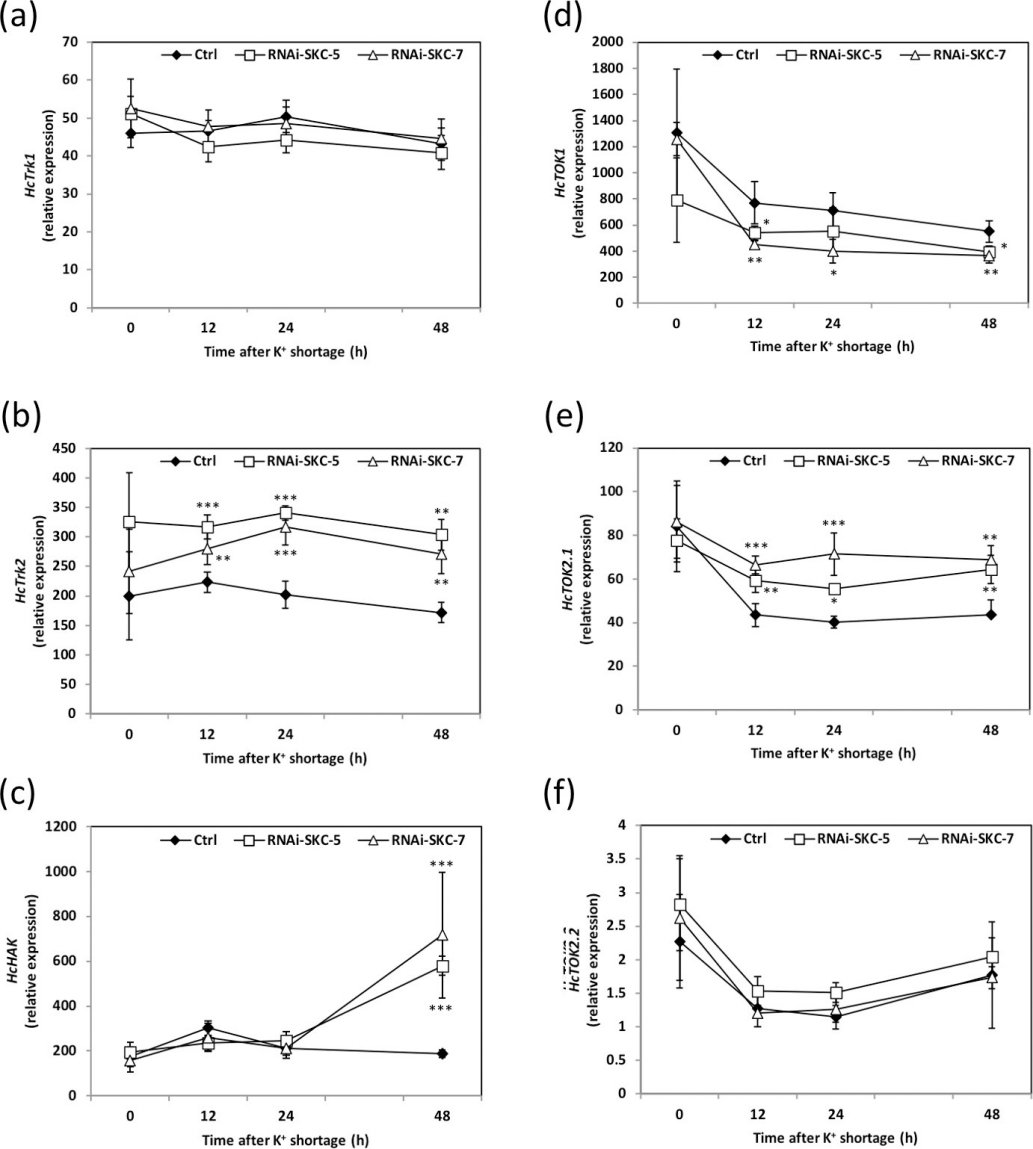
Relative expression of putative K^+^ channels and transporters in *Hebeloma cylindrosporum* control and *HcSKC*-silencing lines. Relative expressions of the K^+^ transporters *HcTrk1* (a), *HcTrk2* (b), *HcHAK* (c) and the K^+^ channels *HcTOK1* (d), *HcTOK2*.*1* (e) and *HcTOK2*.*2* (f) were analyzed in dependence on K^*+*^ shortage in control and RNAi fungal lines. The relative expressions of the K^+^ transporter *HcTrk2* (b) and the K^+^ channels *HcTOK1* (d) and *HcTOK2*.*1* (e) were altered in RNAi-SKC-5 and RNAi-SKC-7 lines in comparison to the transgenic control line (Ctrl) under K^+^ limiting conditions. The relative expressions of *HcTrk1* (a) and *HcHAK* (c) K^+^ transporters, and of the K^+^ channel *HcTOK2*.*2* (f) were not affected by K^+^ shortage in RNAi-SKC-5 and RNAi-SKC-7 lines in comparison to the transgenic control line (Ctrl). Mean values are provided with the standard error (n = 6). Statistical differences were determined using a one-way ANOVA followed by Dunnett’s test relative to Ctrl strain for each data point P < 0.05 (*), P < 0.01 (**) and P < 0.001 (***).

Besides *HcSKC*, six other putative K^+^ transport-related genes can be identified in the *H*. *cylindrosporum* genomic database [4,19]. To figure out whether their expression levels were affected by the downregulation of *HcSKC*, the same fungal samples as those used for K^+^ content measurements were analyzed by qRT-PCR. When grown in K^+^ standard pure culture conditions (SK medium), none of the six targeted K^+^ transport system genes was significantly differentially expressed in the RNAi-SKC lines compared to the control (Fig 5). In contrast, 12, 24 and 48 h after the transfer into the K^+^-free medium, *HcTrk2* and *HcTOK2*.*1* were upregulated and *HcTOK1* was downregulated in the two RNAi-SKC lines (Fig 5B, 5D and 5E). Interestingly, *HcHAK* was upregulated in both RNAi lines only 48 h after the transfer into the K^+^-free medium (Fig 5C). The expression in the transgenic lines of the other two transport systems, *HcTrk1* and *HcTOK2*.*2*, was not significantly affected by the transfer into the K^+^-free medium (Fig 5A–5F). Taken together, these data indicate that the downregulation of *HcSKC* is sufficient to modify the K^+^ homeostasis in *H*. *cylindrosporum* at standard K^+^. However, in conditions of K^+^ shortage, the modification of the expression of three K^+^ transport-related genes compensated for the effect of *HcSKC* downregulation and restored a wild-type K^+^ content in the fungus.

### Downregulation of *HcSKC* affects K^+^ transfer to the host plant at standard K^+^ conditions

**Fig 6:**
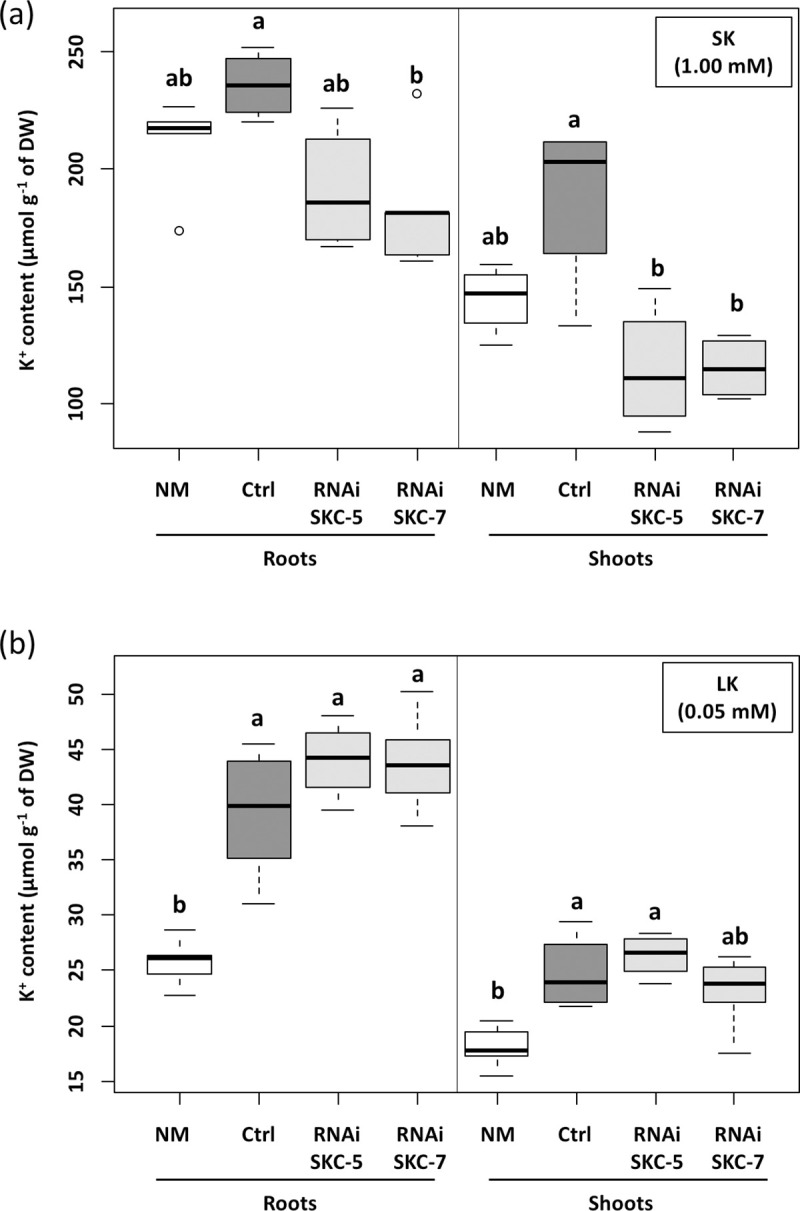
Potassium content in 2-month-old *Pinus pinaster* plants growing alone or in co-culture with *Hebeloma cylindrosporum* under K^+^-sufficient or -deficient conditions. Root and shoot contents were measured in non-mycorrhizal plants (NM) and plants inoculated with fungi transformed with the empty (Ctrl) or *HcSKC*-silencing vectors (RNAi-SKC-5 and RNAi-SKC-7), and growing at standard K^+^ (SK medium, 1 mM K^+^, a) or low K^+^ (LK medium, 0.05 mM K^+^, b). Mean values are provided with the standard error (n = 5–6). Different letters indicate significant differences between treatments according to one-way ANOVA followed by Tukey HSD post-hoc tests (P < 0.05).

To investigate the role of *Hc*SKC in symbiotic conditions, *P*. *pinaster* seedlings were inoculated with control, RNAi-SKC-5, or RNAi-SKC-7 lines, or kept non-inoculated, and grown in SK and LK conditions (Fig 6). Mycorrhization with the control fungal strain (transformed with the empty plasmid) resulted in statistically significant higher K^+^ contents in the plant roots and shoots when grown in LK, but not in SK, conditions. In comparison with these control plants, mycorrhizal association with the *SKC*-silencing fungal lines resulted in a significant decrease of K^+^ contents in shoots and roots in SK conditions, indicating a less cooperative behavior of *H*. *cylindrosporum* that affected K^+^ transfer to *P*. *pinaster* (Fig 6A). Surprisingly, this negative effect of *HcSKC* silencing on K^+^ contents in the host plant was not observed in LK conditions (Fig 6B). Hence, in low K^+^ conditions, the mycorrhizal pathway for plant K^+^ nutrition was not affected by the reduced expression of *HcSKC*. This result suggests that, in this LK condition, other underlying mechanisms are at work to achieve an efficient mycorrhizal K^+^ transport towards the host plant. In other words, *Hc*SKC seems to play a significant role in K^+^ delivery toward the plant in SK conditions (1 mM), and not in conditions of K^+^ shortage (0.05 mM).

## Discussion

As has recently been revealed, K^+^ acquisition in higher fungi seems to be mediated by membrane transport proteins belonging to the Trk/Ktr/HKT and KT/KUP/HAK families [4,43]. In addition, ACU ATPases have been reported as being involved in K^+^ and Na^+^ uptake in *Ustilago maydis*, and more generally envisioned as key players of K^+^ transport in fungi [7,44]. PAT ATPases could emerge as mechanisms for absorption of these cations as well, but they have not been studied thoroughly [45,46]. Regarding outward K^+^ transport systems, yeast and filamentous fungi developed TOK and *Shaker*-like K^+^ channels, from which only some members have been investigated thus far [20,21]. To our knowledge, this work reports the first description of a *Shaker*-like K_V_ channel in higher fungi and highlights its role in both K^+^ homeostasis and mycorrhizal symbiosis.

### *In silico* analysis of *Hc*SKC revealed features from animal *Shaker* K^+^ channels

The initial identification of the *Hc*SKC *Shaker*-like channel was performed from an EST library of *H*. *cylindrosporum* produced in free-living conditions [40]. In this study, one putative *Shaker*-like K^+^ channel was found to be highly expressed (STC no.: 009A07R1.0A1.1), suggesting an important role of this transport system in the fungus. Thanks to the release of *H*. *cylindrosporum* h7 genomic data [19,47,48], *in silico* analyses confirmed the homology of this fungal candidate to animal and plant K_V_ channels (Fig 1). Moreover, phylogenetic analyses provided additional evidence on the proximity of fungal *Shaker* channels to animal ones (Fig 2). The major difference between *Hc*SKC/*Lb*SKC and animal channels is the length of their C-terminal regions. Several studies have evidenced a crucial role of this C-terminal region in the regulation of plant and animal *Shaker* channel activity [49,50]. In plants, it was reported that the C-terminal regions are involved in intracellular K^+^ sensing, heteromerization or voltage-dependent gating mechanisms [51–53]. In animal *Shaker*-type channels, the C-terminus can play multiple roles *e*.*g*. in regulation by β-subunits [54], by voltage [55], or in localization [56]. The fact that the C-terminal domains of *H*. *cylindrosporum* and *L*. *bicolor* are significantly shorter than those of the animal *Shaker* channels (Fig 1) suggests differences in channel activity regulation. Moreover, *Lb*SKC seems to diverge by an additional sequence in the region of the S4 domain (Fig 1B) with unknown function.

### *Shaker*-like channels were lost in Pucciniomycotina, Ustilaginomycotina and Ascomycota phyla

Although well-described in plants and animals, the presence of K_V_ channels in the fungal kingdom has been hypothetical until now. Indeed, no *Shaker* channel gene could be detected in the *Saccharomyces cerevisiae* genome sequence. Our phylogenetic analysis based on many fungal genomes belonging to Basidiomycota and Ascomycota phyla, as well as basal fungi (Cryptomycota, Chytridiomycota, Blastocladiomycota, Zoopagomycota and Mucoromycota phyla) revealed the absence of putative SKC proteins in Pucciniomycotina and Ustilaginomycotina, which are sub-phyla of Basidiomycota, and the entire Ascomycota phylum. Interestingly, one SKC protein was found in *S*. *complicata*, which is related to the Ascomycota phylum. It is, however, worth noting that the affiliation of *S*. *complicata* to Ascomycota remains unclear [57,58], and its SKC protein sequence clusters with those of basal fungi, in agreement with the idea that this species might belong to a more basal phylum than previously thought. Given that *Shaker*-like channels were found in basal fungi and in the most recent subphylum of Badisiomycota (Agaricomycotina), their absence in Pucciniomycotina, Ustilaginomycotina, and Ascomycota strongly suggests that they have been lost in the ancestors of these phyla. This indicates that other transport systems can fill the roles of *Shaker* channels and play a central role in the maintenance of K^+^ homeostasis in these organisms. The survey and analysis of other K^+^ channels found in fungi, such as the TOK channels [4], have been integral in describing other key regulators of K^+^ transport and homeostasis in fungi (*e*.*g*. [20,21]).

### *Hc*SKC cannot be functionally characterized in heterologous systems

Although several attempts using different setups, strategies and heterologous systems were performed, the functional characterization of *H*. *cylindrosporum* and *L*. *bicolor Shaker*-like channels is still missing (S4 Table). Similarly, no conclusive data were obtained after expressing a *HcSKC-EGFP* construct driven by a constitutive or a galactose-inducible promoter in *S*. *cerevisiae* (S2 Fig). This might result from the fact that conditions or proteins required for the expression, function, localization, and regulation of fungal SKC channels were missing in the tested heterologous systems. Recently, the similar failed expression of a zinc transporter from the ECM fungus *Suillus luteus* was reported, showing the risky nature of these experiments [59]. It is well-known that animal and plant *Shaker* channels can physically interact with modulatory β-subunits [60]. For example, rat K_V_1 *Shaker* channels are able to form stable complexes with cytosolic β-subunits (K_V_β), which modify the channel activity [61,62]. In the model plant *Arabidopsis thaliana*, KAB1 is a protein related to animal β-subunits that interacts with the K^+^ channel KAT1 [63]. We identified ten putative β-subunits in the genomic database of *H*. *cylindrosporum* and two of them, *Hc*KCNAB1 and *Hc*KCNAB2, were co-expressed with *HcSKC* in *X*. *laevis* oocytes, resulting in no exogenous current detection. At this stage we cannot conclude whether fungal *Shaker* channels require β-subunits or other types of peptides for their activation. It is known that some compounds can alter the activity of voltage-dependent K^+^ channels, such as human hormones that inhibit K_V_1.3 and K_V_1.5 [64]. Thus, the presence in *X*. *laevis* oocytes of undefined compounds that would inhibit fungal SKC currents might be possible. Another possibility would also be a regulation by other membrane proteins. For instance, in the nematode *Caenorhabditis elegans*, the two-pore K^+^ channel SUP-9 forms a complex with two proteins, UNC-93 (Major Facilitator Superfamily) and SUP-10 (The Potassium Channel Regulatory Protein Sup-10 Family), which coordinate muscle contraction [65]. In *A*. *thaliana*, the homolog of UNC-93 regulates K^+^ translocation from the root to the aerial parts of the plant. Disruption of this gene leads to a phenotype similar to that of knockout mutants of the *AtSKOR Shaker-*like channel, indicating that they may function together, although no physical interactions have yet been observed [66,67]. There is one UNC-93 gene in the genome of *H*. *cylindrosporum* which is induced in ectomycorrhizas, compared to free-living mycelium, and could be a promising candidate to test as a regulator of the activity of *Hc*SKC.

Calcium signaling pathways are well-known regulators of K^+^ transport systems in plants and animals. In plants, calcium mediates signals during biotic interactions and environmental stress, and regulates a series of proteins that can regulate the activity of K^+^ channels through phosphorylation, dephosphorylation, structural modification, and physical interaction [68]. In animals these pathways are different, but there is also evidence of the modulation of K^+^ channel transport by calcium-dependent phosphorylation and dephosphorylation [69]. In filamentous fungi, there are few links between the regulation of K^+^ transport and calcium signaling, probably because most of the studies have been carried on the model Ascomycota yeast *S*. *cerevisiae* [70]. However, the first description of *Shaker*-like K^+^ channels in fungi may lead to the discovery of new pathways. Supporting this hypothesis, a rapid survey of the genome of *H*. *cylindrosporum* yields many putative calcium-regulated genes: three calmodulins (Protein ID 385514, 443591 and 443596), one calcineurin B (444455) and a wide range of Ca^2+^/calmodulin-dependent and serine/threonine protein kinases. It is also tempting to imagine that the most promising heterologous system for the characterization of fungal transport systems would be *S*. *cerevisiae*, which is phylogenetically closer to higher fungi than animal cells. In general, the use of yeast seems more adapted for the characterization of inward transport systems than of outward systems, but so far it has not provided encouraging results in our uptake experiments (S4 Table). Direct transport measurements by the electrophysiological patch-clamp method might provide missing insights in fungal SKC functional properties.

### Potassium homeostasis mediated by *Hc*SKC is a prerequisite for K^+^ allocation to pine

In order to investigate *in vivo* the role of the K_V_ channel SKC in both free-living and ECM conditions, we decided to generate RNAi fungal lines affected in *HcSKC* expression. Analysis of the phenotype of these transgenic fungal lines revealed a close relationship between *HcSKC* expression, K^+^ accumulation in the fungus, and improvement of K^+^ nutrition of the colonized plants. Indeed, *HcSKC* silencing resulted in contrasting phenotypes of both the fungus and the mycorrhizal plant, depending on external K^+^ availability. At 1 mM K^+^, the downregulation of *HcSKC* expression altered K^+^ accumulation in the free-living mycelia and the ECM plants. It should be noted that, in our previous report [12], *H*. *cylindrosporum* had a significant effect on K^+^ allocation to *P*. *pinaster* only in K^+^ limiting conditions and not in sufficient conditions. Such a phenotype is further confirmed in the present report by the comparison between plants inoculated by the control transformed fungus harboring the empty vector and the control non-mycorrhizal plants (Fig 4). However, this does not mean that K^+^ transfer from the fungus to the plant did not occur in standard (SK) conditions. Indeed, the direct uptake of K^+^ from the medium by the root system may be reduced upon mycorrhizal association, and this reduction could be compensated by fungal K^+^ allocation to the plants. Parts of the ECM root system are somehow isolated from the external medium due to a loss of root hairs and to the presence of the fungal mantle surrounding the roots, and thus are dependent on the ECM symbiont to acquire nutrients. Given that *HcSKC* RNAi transformants accumulated more K^+^ than the control strain at standard external K^+^, it may be assumed that, when such transformants were grown in association with *P*. *pinaster*, their reduced expression of *HcSKC* either modified the pool of fungal K^+^ available for the plant and/or affected its symbiotic transfer to host roots. This would result in impaired K^+^ nutrition of the host. It is worth noting that varying external concentrations of K^+^ and Na^+^ did not drastically impact the growth of the RNAi-SKC transgenic fungal lines (S5 Fig). This indicates that the alteration of K^+^ nutrition observed in plants colonized by the RNAi-SKC lines at standard K^+^ cannot be explained by a modification of fungal growth and sensitivity to external conditions, but by the actual downregulation of *HcSKC*. Interestingly, the over-accumulation of K^+^ in the RNAi-SKC transformants was not found to involve the upregulation of putative inward K^+^ transporters, and thus, might result from a decrease in K^+^ efflux from the fungus. In contrast, in low K^+^ conditions, three other putative K^+^ transport systems were up- or downregulated in the RNAi lines. We postulate that the altered expression of *HcTrk2*, *HcHAK*, *HcTOK1* and *HcTOK2*.*1* restored the overall fungal K^+^ transport activities and homeostasis, as suggested by the recovery of the fungal K^+^ content observed in free-living conditions (Fig 4). This could explain the restoration of the K^+^ contents displayed by the mycorrhizal plants, suggesting that efficient ectomycorrhiza-mediated K^+^ nutrition occurred again. Another hypothesis, that *Hc*SKC is involved in K^+^ movements from/to the vacuole, cannot be completely excluded, even though only plasma membrane localization of this type of ion channel has been described in any organism so far. Subcellular localization at either the plasma membrane or at the tonoplast should allow us to fully appreciate the role of *Hc*SKC, regarding its manifest importance in *H*. *cylindrosporum* biology under axenic and symbiotic conditions.

To summarize, homology of *Hc*SKC with animal outward K_V_ channels and the observed K^+^ nutrition-related mycorrhizal and fungal phenotypes lead to the simplest hypothesis: that the fungal *Shaker*-like channel *Hc*SKC may be involved in K^+^ efflux from fungal cells, allowing K^+^ translocation towards the host plant. Thus, the role played by *Hc*SKC in K^+^ homeostasis in *H*. *cylindrosporum* seems crucial for fungal K^+^ availability and/or release to the host plant.

## Supporting information

S1 Fig Alignment of *Hc*SKC subunit of *Hebeloma cylindrosporum* with *Rattus norvegicus Rn*K_V_2.1. Structure-based sequence alignment and sequence conservation between *Hc*SKC and *Rn*K_V_2.1 K^+^ channel from *Rattus norvegicus*. The GYGE motif of the pore domain was highlighted in green.
(TIF)

S2 Fig Attempts to localize *Hc*SKC with a C-terminal EGFP fusion in *Saccharomyces cerevisiae*. The *HcSKC-EGFP* construct was inserted into the pYES2 vector, with galactose-induced expression (a,b), or into the pFL61 vector, under the PGK constitutive promoter (c,d). None of these attempts yielded a clear localization pattern of *Hc*SKC, probably due to misprocessing of the protein product.
(TIF)

S3 Fig Analysis of EGFP expression under control of the *HcSKC* promoter in *Hebeloma cylindrosporum*. EGFP expression was observed in fungal lines harboring the EGFP cassette under control of the promoters of *HcSKC* (pPZP-PSKC1-E). Fungal isolates were grown on YMG medium for 2–3 weeks before analysis. (a) bright field image (b) EGFP image (GFP filter 505–530 nm). Scale bar: 100 μm.
(TIF)

S4 Fig Determination of *Hebeloma cylindrosporum* silencing *HcSKC* lines. The relative expression of *HcSKC* was determined in empty vector (Ctrl), RNAi-SKC-5 and RNAi-SKC-7 fungal lines by qRT-PCR. Mean values are provided with the standard deviation (n = 6). Statistical differences were evaluated using the Student’s test with respect to E.V. strain (**, P < 0.01).
(TIF)

S5 Fig Biomass of *Hebeloma cylindrosporum HcSKC*-silencing lines under high and low potassium and sodium regimes. Dry weights of empty vector (Ctrl), RNAi-SKC-5 and RNAi-SKC-7 fungal lines were determined after 28 days of culture in media containing high (+K and +Na) or low concentration (-K and -Na) of potassium (K^+^) and sodium (Na^+^), respectively. +K corresponds to 1 mM of K^+^, -K to 0.05 mM of K^+^, +Na to 1 mM of Na^+^, and -Na to 0.2 mM of Na^+^. Full recipes are provided in S1 Table. Mean values are provided with the standard deviation (n = 4–6). Different letters indicate significant differences between treatments according to one-way ANOVA followed by Tukey HSD post-hoc tests (P < 0.05).
(TIF)

S1 Table Primer list. Restriction enzyme sites indicated in primer name, were underlined in the corresponding 5’-3’ sequence.
(TIF)

S2 Table Protocol of media used to assess the impact of high and low potassium and sodium conditions on the biomass of transgenic fungal strains Ctrl, RNAi-SKC-5 and RNAi-SKC7. Final concentrations of components constituting the +K/-Na, +K/+Na, -K/-Na, and -K/+Na media used in S5 Fig were presented in this table. pH was adjusted at 5.5 with Ca(OH)2 before autoclave.
(TIF)

S3 Table Sequences used in *in silico* and phylogenetic analyses. (XLS)

S4 Table Attempts for functional characterization of the *Shaker*-like channels *Hc*SKC(strain h7), HcSKC1&2 (strain h1) from *Hebeloma cylindrosporum*, and *Lb*SKC from *Laccaria bicolor*, respectively. Whole-cell current recordings in *Xenopus* oocytes expressing *Hc*SKC alone or with two β-subunits or a kinase as well as expression of *Lb*SKC did not give evidence for K^+^-dependent currents. Growth of a triple yeast mutant PLY246 (*Δtrk1Δtrk2Δtok1*; Bertl *et al*., 2003) was not complemented by expression of *Hc*SKC.
(TIF)
