# Supplementary figures and images for: Fungal Shaker-like channels beyond cellular K+ homeostasis: A role in ectomycorrhizal symbiosis between Hebeloma cylindrosporum and Pinus pinaster
Source: PLoS One. 2020 Nov 20;15(11):e0242739. doi: 10.1371/journal.pone.0242739 (PMC7678990; doi:10.1371/journal.pone.0242739)

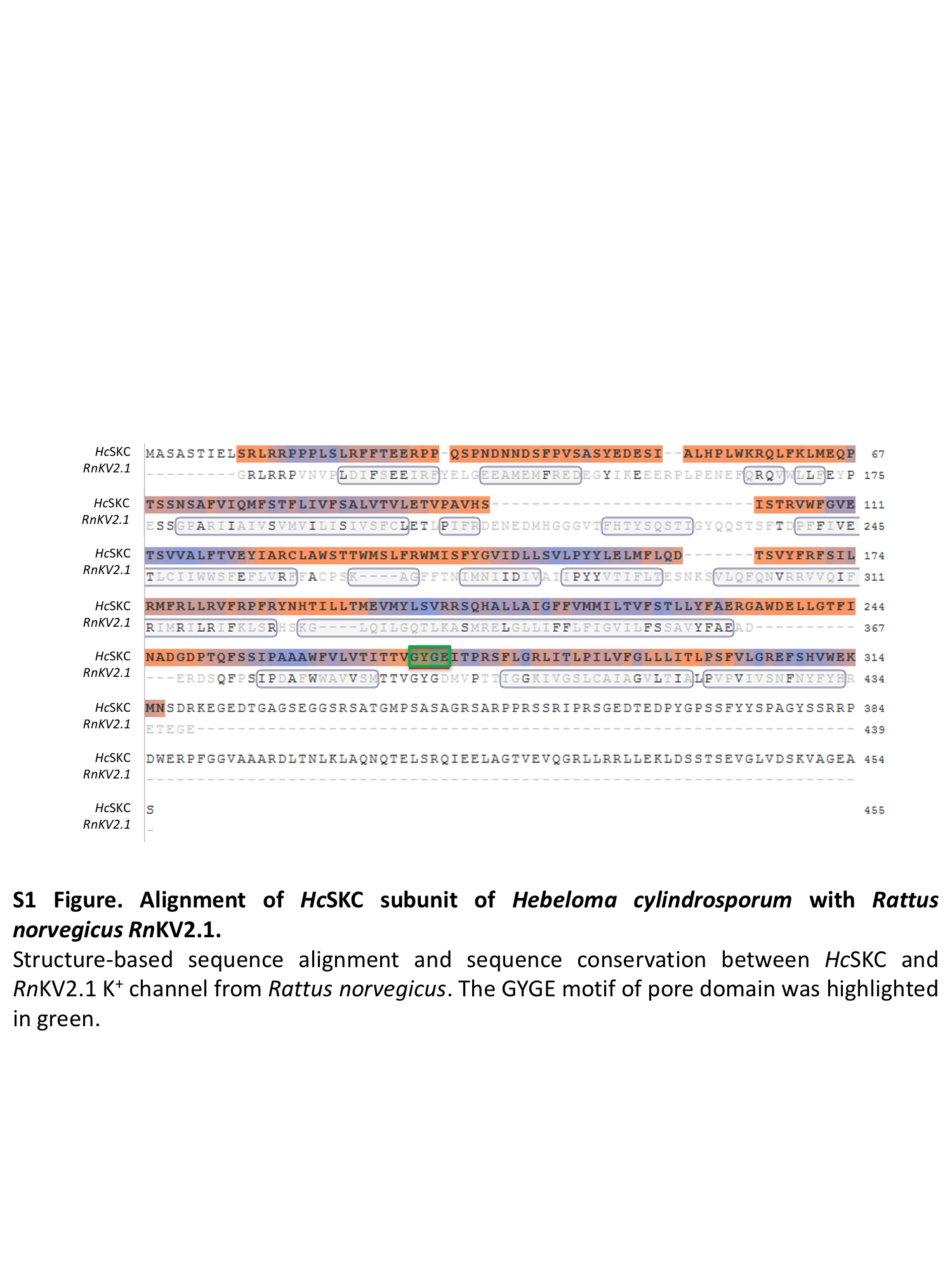

Supplement: S1 Fig — Structure-based sequence alignment and sequence conservation between HcSKC and RnKV2.1 K+ channel from Rattus norvegicus. The GYGE motif of the pore domain was highlighted in green. (TIF) [file pone.0242739.s001.tif]

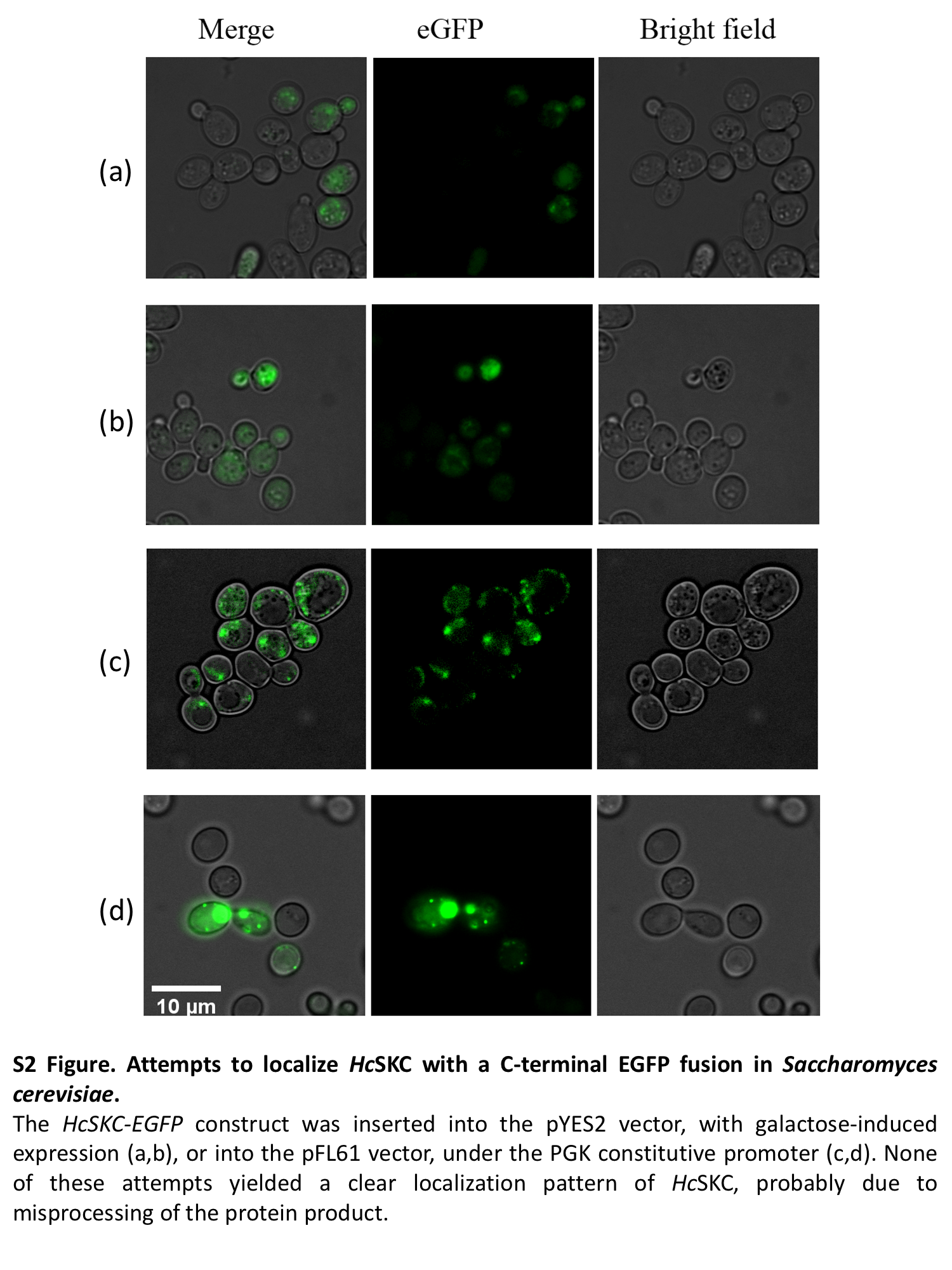

Supplement: S2 Fig — The HcSKC-EGFP construct was inserted into the pYES2 vector, with galactose-induced expression (a,b), or into the pFL61 vector, under the PGK constitutive promoter (c,d). None of these attempts yielded a clear localization pattern of HcSKC, probably due to misprocessing of the protein product. (TIF) [file pone.0242739.s002.tif]

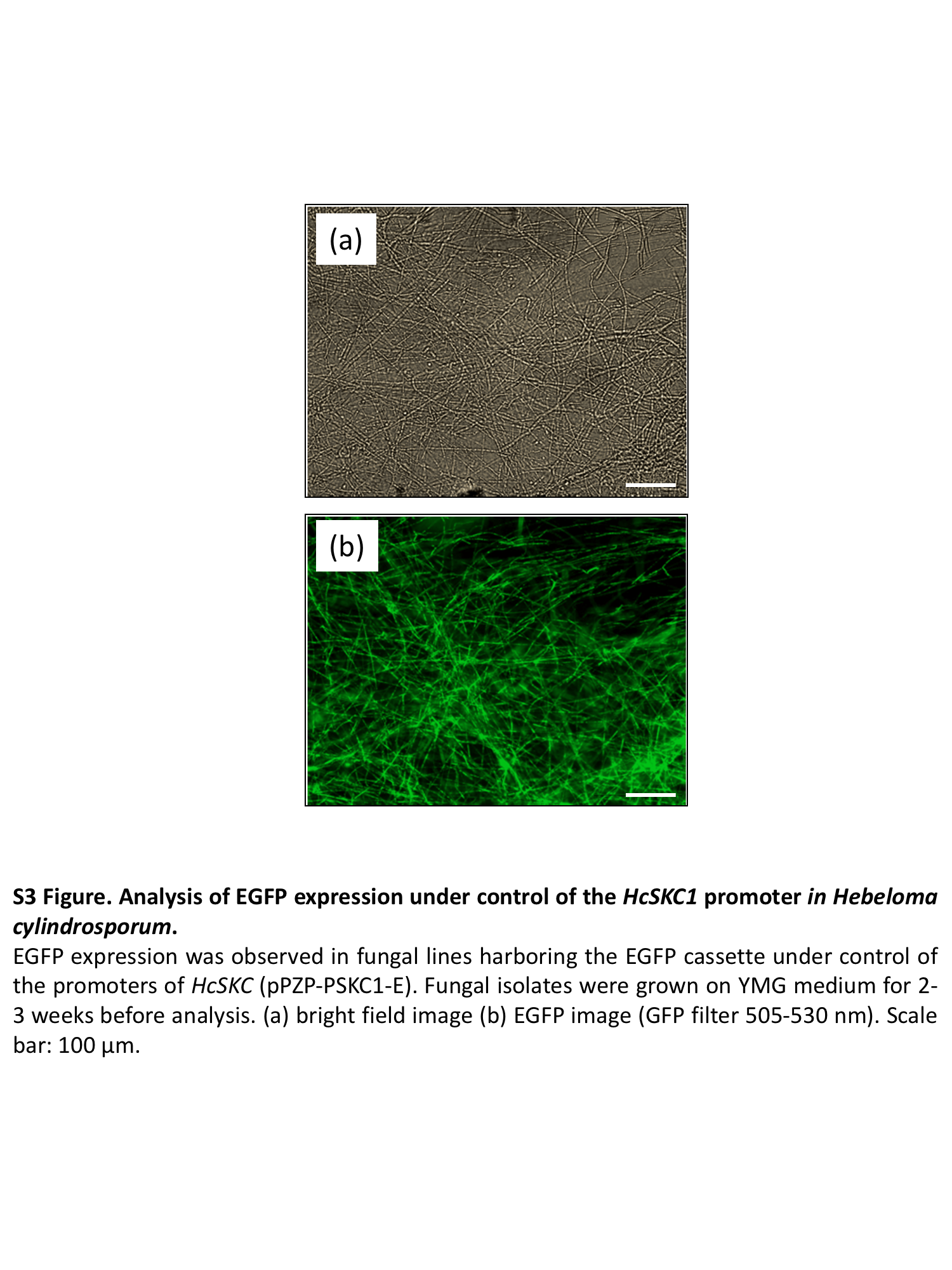

Supplement: S3 Fig — EGFP expression was observed in fungal lines harboring the EGFP cassette under control of the promoters of HcSKC (pPZP-PSKC1-E). Fungal isolates were grown on YMG medium for 2–3 weeks before analysis. (a) bright field image (b) EGFP image (GFP filter 505–530 nm). Scale bar: 100 μm. (TIF) [file pone.0242739.s003.tif]

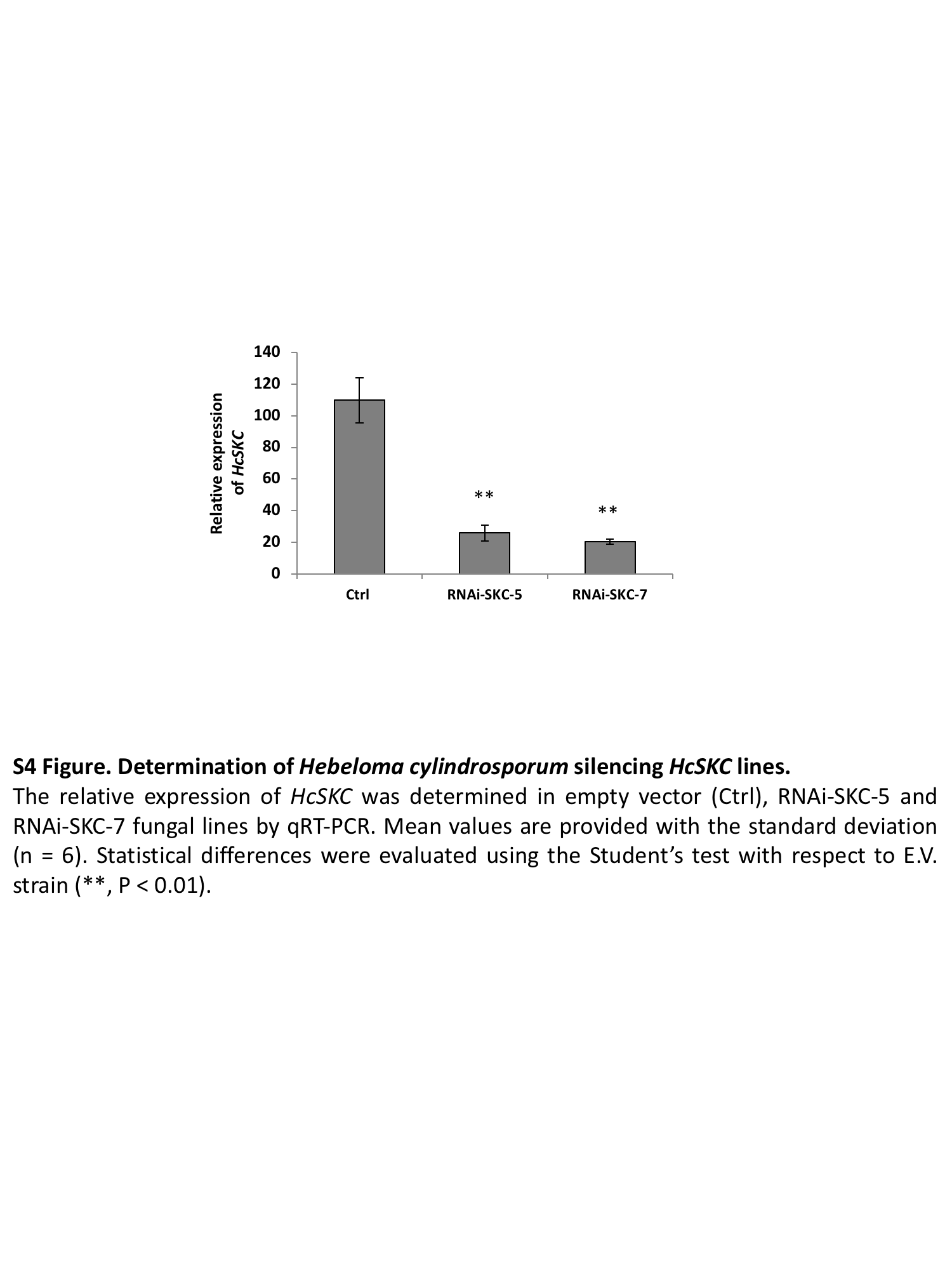

Supplement: S4 Fig — The relative expression of HcSKC was determined in empty vector (Ctrl), RNAi-SKC-5 and RNAi-SKC-7 fungal lines by qRT-PCR. Mean values are provided with the standard deviation (n = 6). Statistical differences were evaluated using the Student’s test with respect to E.V. strain (**, P < 0.01). (TIF) [file pone.0242739.s004.tif]

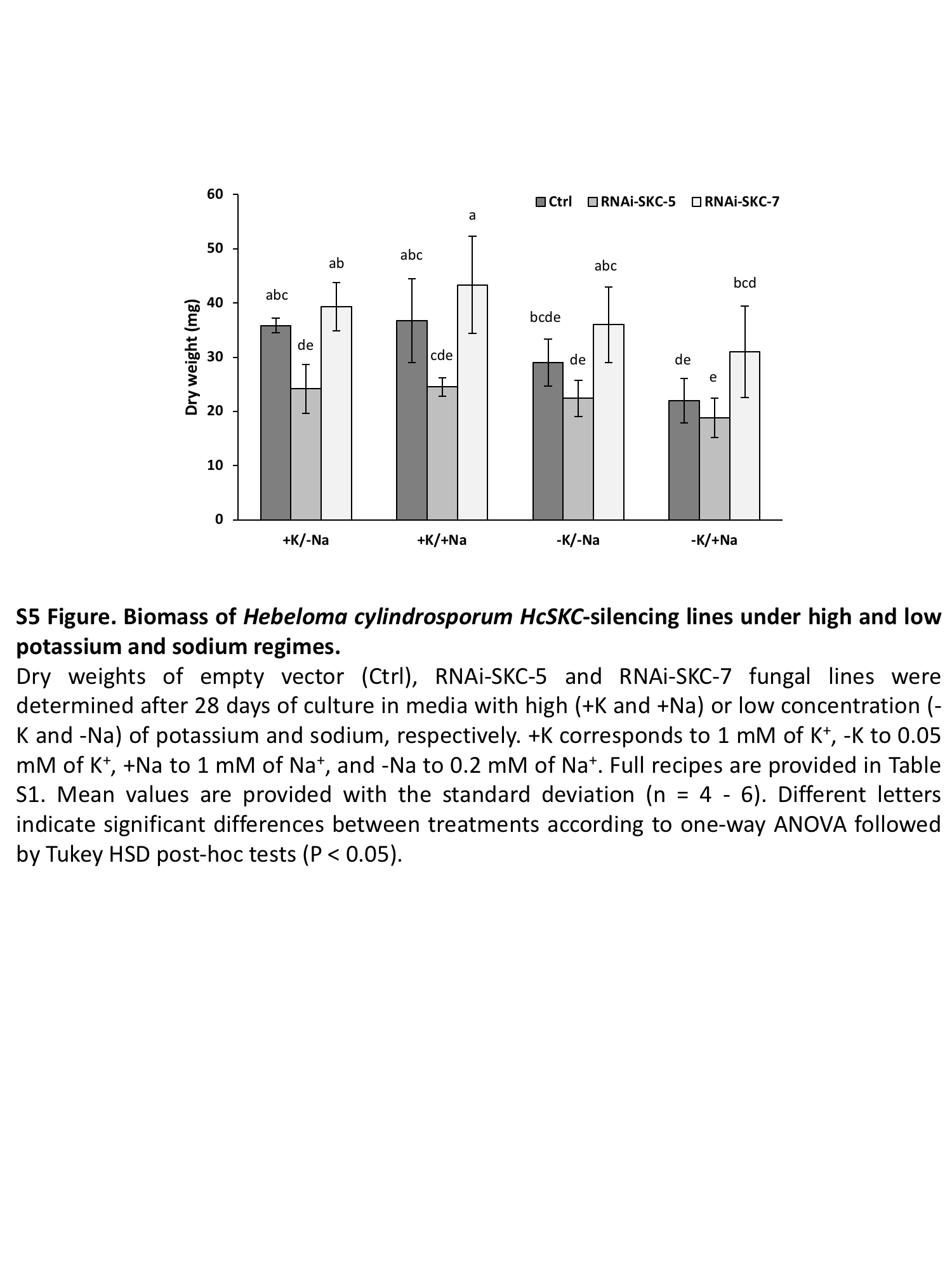

Supplement: S5 Fig — Dry weights of empty vector (Ctrl), RNAi-SKC-5 and RNAi-SKC-7 fungal lines were determined after 28 days of culture in media containing high (+K and +Na) or low concentration (-K and -Na) of potassium (K+) and sodium (Na+), respectively. +K corresponds to 1 mM of K+, -K to 0.05 mM of K+, +Na to 1 mM of Na+, and -Na to 0.2 mM of Na+. Full recipes are provided in S1 Table. Mean values are provided with the standard deviation (n = 4–6). Different letters indicate significant differences between treatments according to one-way ANOVA followed by Tukey HSD post-hoc tests (P < 0.05). (TIF) [file pone.0242739.s005.tif]

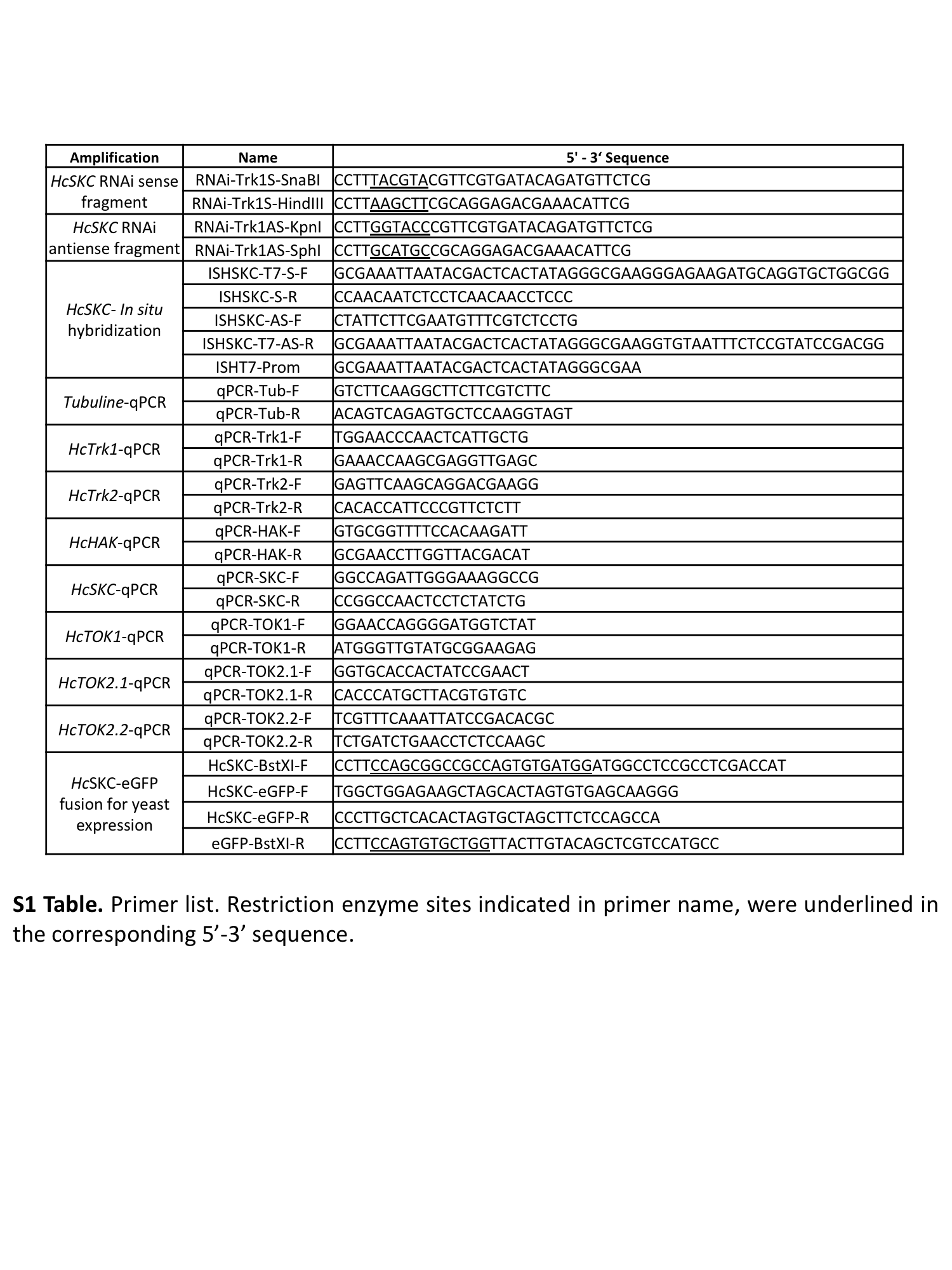

Supplement: S1 Table — Restriction enzyme sites indicated in primer name, were underlined in the corresponding 5’-3’ sequence. (TIF) [file pone.0242739.s006.tif]

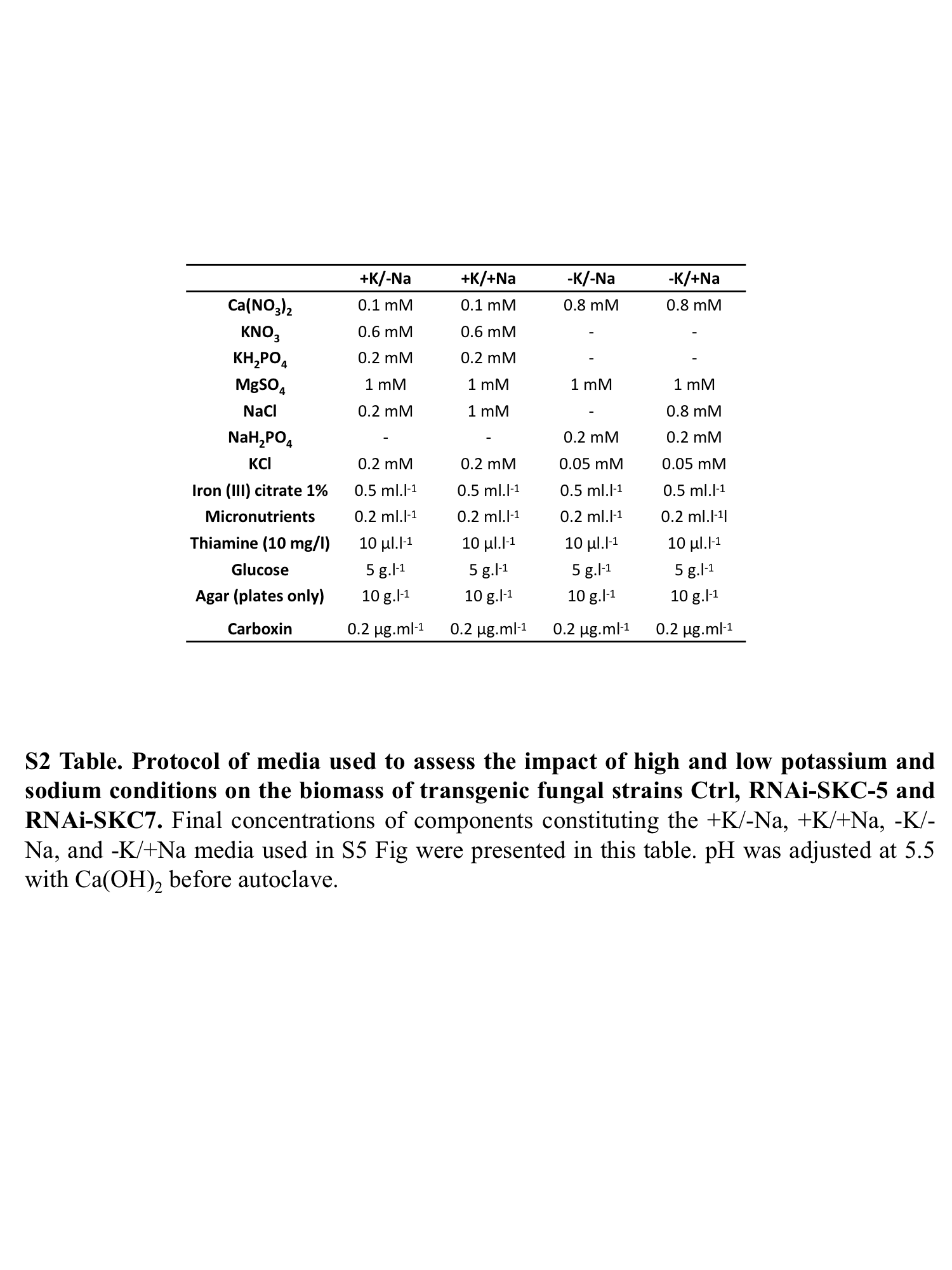

Supplement: S2 Table — Final concentrations of components constituting the +K/-Na, +K/+Na, -K/-Na, and -K/+Na media used in S5 Fig were presented in this table. pH was adjusted at 5.5 with Ca(OH)2 before autoclave. (TIF) [file pone.0242739.s007.tif]

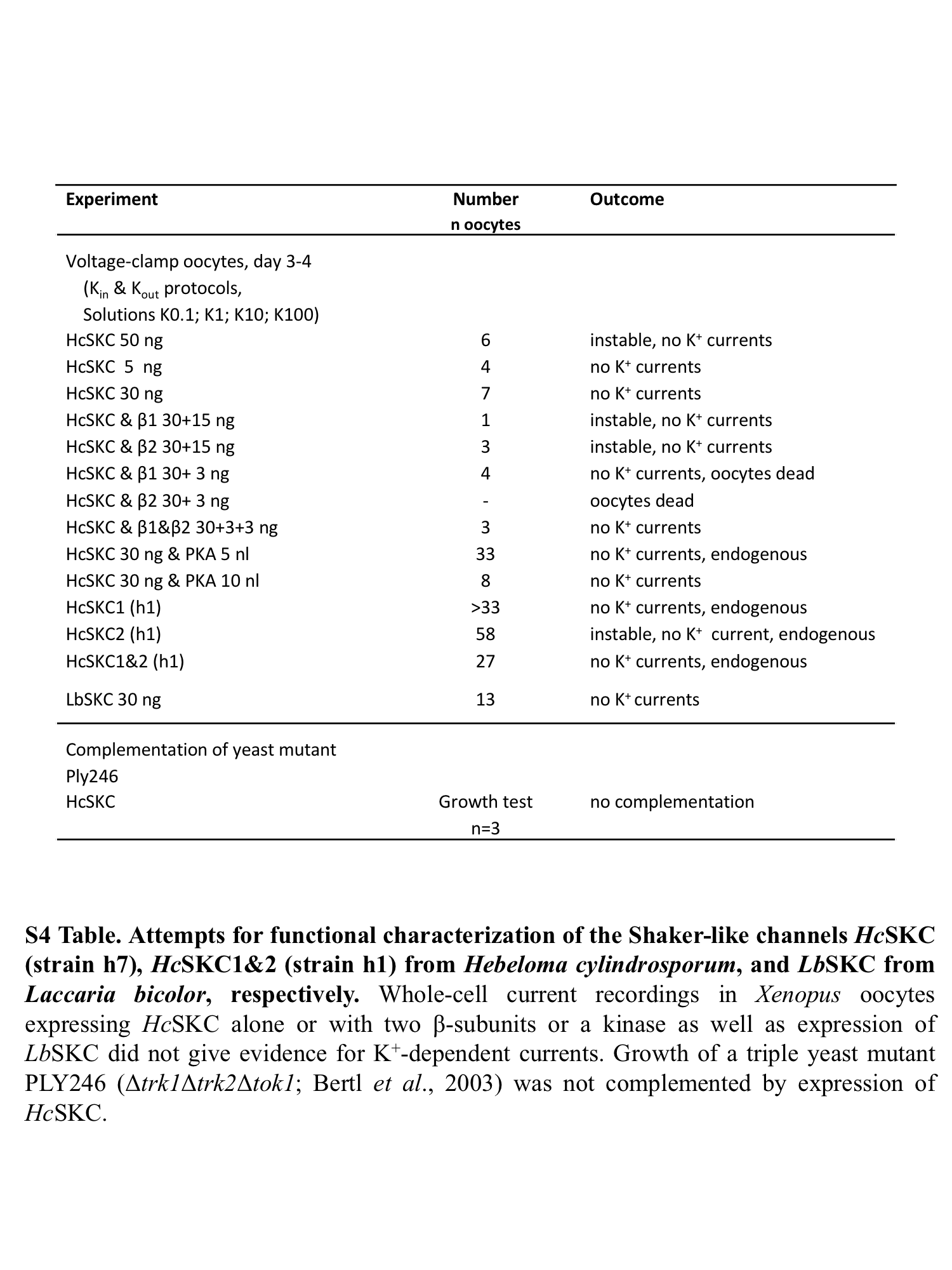

Supplement: S4 Table — Whole-cell current recordings in Xenopus oocytes expressing HcSKC alone or with two β-subunits or a kinase as well as expression of LbSKC did not give evidence for K+-dependent currents. Growth of a triple yeast mutant PLY246 (Δtrk1Δtrk2Δtok1; Bertl et al., 2003) was not complemented by expression of HcSKC. (TIF) [file pone.0242739.s009.tif]
